# Eu- or hypoglycemic ketosis and ketoacidosis in children: a review

**DOI:** 10.1007/s00467-023-06115-5

**Published:** 2023-08-16

**Authors:** Martina Meoli, Sebastiano A. G. Lava, Gabriel Bronz, Barbara Goeggel-Simonetti, Giacomo D. Simonetti, Ilaria Alberti, Carlo Agostoni, Mario G. Bianchetti, Martin Scoglio, Stefano A. Vismara, Gregorio P. Milani

**Affiliations:** 1 Family Medicine Institute, Università Della Svizzera Italiana, 6900 Lugano, Switzerland; 2 Pediatric Cardiology Unit, Department of Pediatrics, Centre Hospitalier Universitaire Vaudois and University of Lausanne, Lausanne, Switzerland; 3 Pediatric Institute of Southern Switzerland, Ospedale San Giovanni, Bellinzona, Switzerland; 4 Pediatric Unit, Fondazione IRCCS Ca’ Granda Ospedale Maggiore Policlinico, Milan, Italy; 5 Department of Clinical Sciences and Community Health, Università Degli Studi Di Milano, Milan, Italy

**Keywords:** Ketosis, Ketone bodies, Metabolic acidosis, Diet, Protein, Glucose

## Abstract

The last decade has been characterized by exciting findings on eu- or hypoglycemic ketosis and ketoacidosis. This review emphasizes the following five key points: 1. Since the traditional nitroprusside-glycine dipstick test for urinary ketones is often falsely negative, the blood determination of β-hydroxybutyrate, the predominant ketone body, is currently advised for a comprehensive assessment of ketone body status; 2. Fasting and infections predispose to relevant ketosis and ketoacidosis especially in newborns, infants, children 7 years or less of age, and pregnant, parturient, or lactating women; 3. Several forms of carbohydrate restriction (typically less than 20% of the daily caloric intake) are employed to induce ketosis. These ketogenic diets have achieved great interest as antiepileptic treatment, in the management of excessive body weight, diabetes mellitus, and in sport training; 4. Intermittent fasting is more and more popular because it might benefit against cardiovascular diseases, cancers, neurologic disorders, and aging; 5. Gliflozins, a new group of oral antidiabetics inhibiting the renal sodium-glucose transporter 2, are an emerging cause of eu- or hypoglycemic ketosis and ketoacidosis. In conclusion, the role of ketone bodies is increasingly recognized in several clinical conditions. In the context of acid–base balance evaluation, it is advisable to routinely integrate both the assessment of lactic acid and β-hydroxybutyrate.

## Introduction

Available reviews on ketosis primarily focus on the association with diabetes and starvation and the role of ketone bodies as an efficient fuel source [1–3]. Recent research has vastly increased the understanding of ketosis and ketoacidosis. To bridge the gap between scientific evidence and clinical practice, this review aims to evaluate and incorporate new insights about mechanisms, diagnosis, and consequences of ketosis and ketoacidosis.

## Historical background

It has been known for almost two centuries that patients with diabetes mellitus may present with an increased depth and rate of breathing, generally referred to as Kussmaul breathing pattern [4, 5]. Breath and urine of these patients show a peculiar fruity odor, often referred to as the odor of acetone (the Latin terms “odor acetoni” and “foetor acetonemicus” (or acetonicus) are also used). Furthermore, the urine of these patients was found to give, with iron perchloride (Gerhardt reaction), a purplish-red color that is due to ketone bodies [6, 7]. It has also been known for a long time that the Kussmaul breathing pattern, fruity odor, and a positive Gerhardt reaction may occur during periods of low food intake [6, 7].

Ketone bodies were long thought to be an undesirable product of fat metabolism [3]. Only after the Second World War were ketone bodies recognized as normal metabolites, which are utilized by tissues including brain as a major fuel in healthy infants and during energy restriction [3, 8–10]. It has also been known since ancient times that fasting [8, 9] and, subsequently, ketosis could treat epilepsy (as suggested among others in the Bible, chapter 17 of the Gospel of St. Matthew: “this disease [= epilepsy] does not leave but by fasting”).

## Ketone bodies and their assessment

The terms ketone acids, ketone bodies, and, simply, ketones are mostly interchangeably used (including in this review) to denote acetoacetate, β-hydroxybutyrate, and acetone [9, 10]. Acetoacetate (and acetoacetic acid; acidity constant pK_a_ ≈ 3.58) and β-hydroxybutyrate (and β-hydroxybutyric acid; acidity constant pK_a_ ≈ 4.82) are acid and dominant [9, 11]. Acetone, the less important ketone body, is spontaneously formed from acetoacetate but is not an acid (pK_a_ ≈ 19.2). Hence, some experts use the term ketones exclusively for acetoacetate and β-hydroxybutyrate [3, 9, 11].

Currently available dipstick tests for ketones detect acetoacetate and, to a lesser extent, acetone in urine using a reaction involving nitroprusside and glycine known as the Rothera reaction [12, 13]. However, this simple and cheap test does not detect β-hydroxybutyrate [12, 13]. In everyday practice, there is a high rate of false-negative tests [12, 13]. False-positive results occasionally occur on medication with captopril, mesna, or penicillamine [12, 13]. Despite these drawbacks, this test continues to be the predominantly utilized method.

For a comprehensive assessment of ketone body status, acetone, acetoacetate, and β-hydroxybutyrate should be separately measured in blood and subsequently summed as total ketone concentration. As acetone is not acid, acetoacetate chemically unstable, and β-hydroxybutyrate the dominant ketone body,^1^ most experts nowadays consider that the level of β-hydroxybutyrate in blood provides an accurate and direct estimation of the individual’s ketone body status [11, 14]. Compelling research findings, yet to be incorporated into everyday clinical routine, indicate that there are now viable methods to precisely measure β-hydroxybutyrate levels in both venous and capillary blood [14]. This approach overcomes the challenges associated with the urinary nitroprusside-glycine test (Rothera reaction).

## Metabolism of ketone bodies

**Fig. 1:**
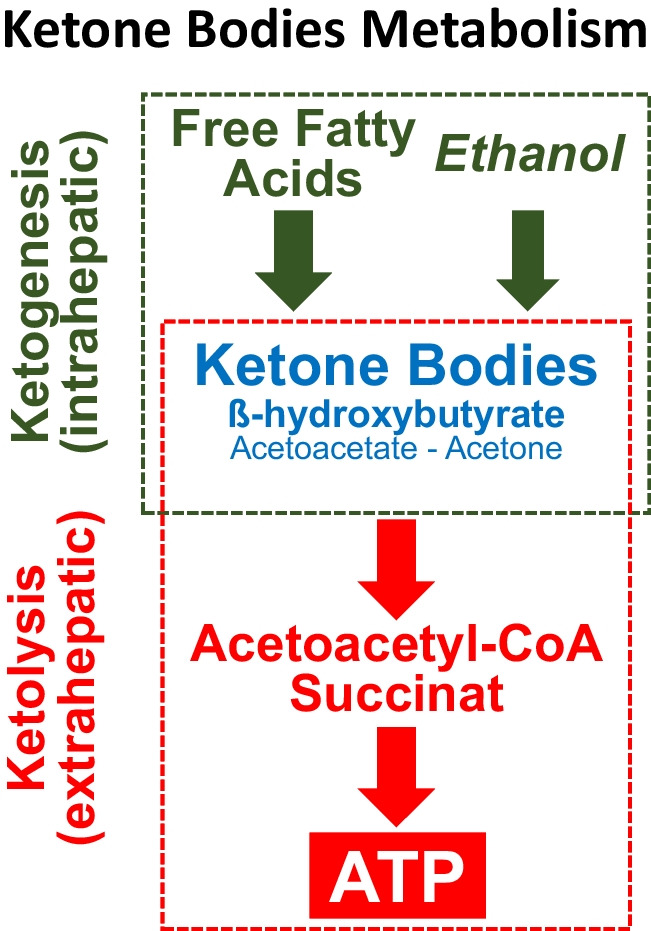
Ketone body generation (ketogenesis) and utilization (ketolysis). The activity of enzymes implicated in the formation of ketone bodies is stimulated by insulin deficiency, fasting, and consumption of a high-fat intake. While acetoacetate and β-hydroxybutyrate are readily metabolized by extrahepatic tissues, acetone is difficult to metabolize in vivo and is to a large extent volatilized in the lungs. Ethanol is a further potential precursor of ketone bodies

The metabolism of ketone bodies [3, 9, 11, 15, 16] includes ketogenesis, in which the liver mitochondria transform free fatty acids into acetoacetate and β-hydroxybutyrate, and ketolysis, in which ketones are converted into useful energy by the mitochondria of many extrahepatic tissues (Fig. 1). Acetoacetate and β-hydroxybutyrate may also be metabolized from ethanol (see below).

The term ketosis denotes an upregulated synthesis of ketone bodies [3, 9, 11, 15]. On the other hand, [hyper]ketonemia denotes a relevant increase in circulating ketones (the β-hydroxybutyrate level in blood is normally < 0.5 mmol/L). Very often, however, the terms ketosis and [hyper]ketonemia are interchangeably used [9, 11, 15]. Finally, the term β-hydroxybutyrate ketosis is sometimes employed instead of that of ketosis, highlighting the prominence of this ketone body in this condition [9, 11, 15].

A molecule of glucose [9, 11, 15] produces 36–38 molecules of adenosine triphosphate (however, current data support the notion that the yield is about 30–32). By contrast, acetoacetate and β-hydroxybutyrate yield 26 and 21 molecules of adenosine triphosphate, respectively [9, 10, 15, 16]. Since ketones are, together with glucose, the principal energy source in humans, fasting is the most recognized physiological cause of ketogenesis and ketolysis [3, 6, 9, 11].

The concentration of circulating ketone bodies is slightly higher in healthy newborn babies as compared to older children and adults. This is likely related to the high-fat concentration (approximately 35–40 g/L) in human and cow milk. Nonetheless, there is usually no ketonuria in healthy newborns [10, 16, 17].

β-hydroxybutyrate is not only an energy carrier but also plays crucial roles in cellular signaling. These effects may help explain the beneficial advantages of ketogenic diets [18].

## Causes of eu- or hypoglycemic ketosis

**Fig. 2:**
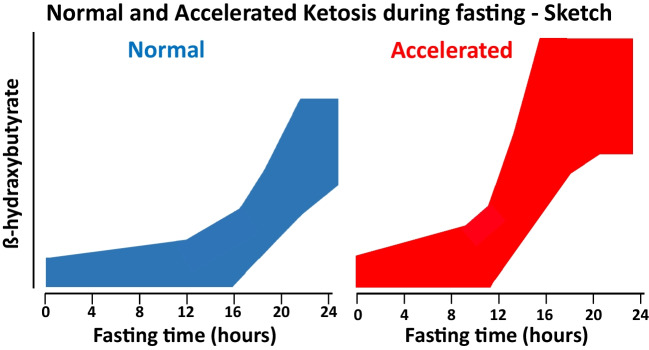
Sketch depicting circulating β-hydroxybutyrate as a function of fasting in adults and children ≥ 8 years of age (left panel; normal fasting-associated ketosis) and in stages of life that predispose to accelerated fasting-associated ketosis such as newborns, infants, children 7 years of age or less (especially during infectious diseases), and pregnant, parturient, or lactating women (right panel)

Fasting is the most recognized cause of eu- or hypoglycemic ketosis and ketoacidosis [3, 16, 17]. In healthy adults, adolescents, and older children, ketosis usually occurs approximately after 12–18 h of fasting (“normal ketosis”; Fig. 2). In the mentioned age groups, the β-hydroxybutyrate concentration is approximately 1 mmol/L after fasting for 12–18 h. If fasting persists, its concentration continues to rise and peaks after 20–30 days at 6–8 mmol/L. At this ketone body concentration, the rate of hepatic ketogenesis matches the cerebral, muscular, and renal energy requirements (plus a small degree of ketonuria) [3, 9, 11]. The time interval is shorter than 12–18 h and the β-hydroxybutyrate level is higher in subjects with low glycogen stores such as newborns and infants (“accelerated ketosis”; Fig. 2). This tendency is also observed in children 7 years of age or less over the course of acute infections, which are characterized by catabolism exceeding anabolism [3, 9, 11, 16, 17]. Pregnancy [19, 20], parturition [21], lactation [22, 23], and prolonged exercise [24] are further common physiological causes of ketosis.

- In the last decade, ketogenic diets (also referred to as “keto diets”), which involve eating foods that are low in sugars (typically less than 20% of the daily caloric intake), have achieved great interest as antiepileptic treatment, in the management of excessive body weight, diabetes, and in sport training [25–30]. Considering the heterogeneous terminology in the literature, the total caloric intake is currently used to subdivide these diets into three groups: isocaloric ketogenic diet (e.g., Atkins and Dukan diet), low-calorie ketogenic diet, or very-low-calorie ketogenic diet. While both the Atkins and Dukan diets restrict sugar consumption, the Dukan diet, unlike the Atkins diet, also limits fat intake [31, 32]. Because these regimens are stringent and difficult to maintain in practice, alternatives have been developed, in which “fats” are provided as exogenous medium-chain triglycerides (as octanoic or decanoic acid) or ketones as a substitute method of inducing therapeutic ketosis without the need for a rigorous dietary regimen [33, 34]. Intermittent fasting, which induces transient ketosis, has emerged as a popular approach to weight loss. Beyond its weight loss benefits, this practice has been suggested to have potential therapeutic implications for a range of disorders including diabetes mellitus, blood pressure, cardiovascular disease, cancers, and neurologic disorders. This practice has been an age-old health habit that has been observed throughout history [35, 36].
- Cyclic vomiting syndrome is characterized by recurrent bouts of vomiting and is considered a variant of migraine headache. In the past, it was thought to affect exclusively children [37]. Since there is no specific test, it is a diagnosis of exclusion after multiple evaluations for the same recurring symptoms [38, 39]. Noteworthy, there is a broad metabolic differential to consider including intermittent porphyria, fatty acid oxidation disorders, mitochondrial disorders, organic acidemias, and urea cycle defects [15, 38, 39]. In cyclic vomiting syndrome, early symptoms are treated with a triptan-family drug [15, 38, 39]. However, triptans are not approved for subjects 12 years or less of age. The management of acute vomiting episodes is addressed below.
- Alcohol abuse is currently pervasive and endemic among adolescents worldwide [40]. Ketosis may develop after binge ethanol ingestion, most frequently in subjects with preexisting abuse [7, 15, 41–43]. In the liver, ethanol is metabolized to acetoacetate and reduced nicotinamide adenine dinucleotide, and, subsequently, to β-hydroxybutyrate. Excess amounts of reduced nicotinamide adenine play a central role in the transformation of acetoacetate to β-hydroxybutyrate. Since reduced nicotinamide adenine also inhibits gluconeogenesis, alcoholic ketosis may be associated with hypoglycemia. Although mild ketosis can develop during ethanol ingestion, severe ketosis and ketoacidosis usually occur many hours after ethanol ingestion has ceased. For this reason, in alcoholic ketoacidosis, ethanol level is habitually low or undetectable at presentation [7, 15, 41–43]. Because in this condition up to 90% of ketones are in the form of β-hydroxybutyrate, the nitroprusside-glycine urine dipstick is frequently negative. Poisoning with isopropanol causes features that resemble those observed with binge ethanol ingestion [7, 15, 41–43]. Poisoning with other alcohols, glycols, and ketones can also lead to ketoacidosis [15].

Further rather rare causes of ketosis include some congenital disorders of intermediary metabolism [16, 38, 39], severe untreated hyperthyroidism [44], acute salicylate intoxication [15], and medication with gliflozins [1, 45]. Ketoacidosis, often associated with hypoglycemia, lactic acidosis, and sometimes also with hyperammonemia, occurs in subjects affected by congenital disorders of intermediary metabolism including propionic acidemia, methylmalonic acidemia, maple syrup urine disease, isovaleric acidemia, 3-methylcrotonylglycinuria, glutaric aciduria type I, biotinidase deficiency, and holocarboxylase synthetase deficiency. Affected subjects mostly present acutely in the neonatal period with nonspecific symptoms and signs [16, 38, 39]. More rarely, they can present later. An exhaustive description of these conditions is beyond the scope of this review.

**Fig. 3:**
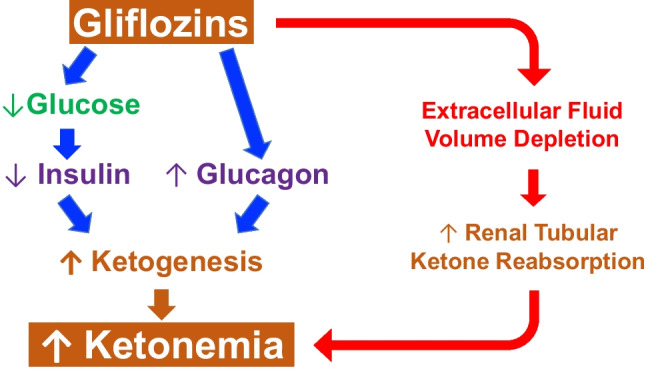
Mechanisms likely underlying euglycemic ketosis following treatment with gliflozins. First, gliflozins lower blood glucose (by enhancing glucosuria) and subsequently insulin, which stimulates ketogenesis (left panel). In addition, gliflozins induce the secretion of glucagon, which also likely contributes to the formation of ketone bodies (left panel). Finally, gliflozins induce osmotic diuresis and reduce the extracellular volume. It is assumed that volume depletion increases renal tubular ketone body reabsorption and thereby further enhances hyperketonemia (right panel)

Salicylate intoxication results in a metabolic acidosis concomitantly caused by accumulation of salicylic acid, lactic acid, and β-hydroxybutyric acid [15]. Gliflozins, which inhibit the renal sodium-glucose transporter 2, enhance glucosuria thereby lowering blood glucose level. They are prescribed in type 2 and, less frequently, type 1 diabetes mellitus. These drugs are also used to treat heart failure both in diabetic and nondiabetic patients. However, gliflozins predispose to euglycemic ketosis [45]. It is postulated that two main factors account for this peculiar form of ketosis. First, gliflozins enhance glucosuria, lower blood glucose and subsequently insulin, therefore stimulating ketogenesis. In addition, gliflozins enhance the secretion of glucagon, which also contributes to the formation of ketone bodies (Fig. 3). Gliflozins cause osmotic diuresis and reduce the extracellular volume. It has been therefore suggested that volume depletion increases renal tubular ketone body reabsorption and thereby further enhances the tendency to hyperketonemia. Although a mild increase in circulating ketones during gliflozin therapy is common, relevant euglycemic ketosis induced by gliflozins, and even more so ketoacidosis, is rare and usually occurs in the presence of further factors such as concomitant insulin therapy, type 1 diabetes mellitus, low carbohydrate intake, acute intercurrent illness, pregnancy, or acute ethanol ingestion [45]. Euglycemic ketoacidosis is exquisitely rare in children [46].

## Effects of ketosis

Ketosis notoriously reduces the perception of hunger [47]; induces nausea, vomiting, stomach pain, or flu-like symptoms (Table 1); and predisposes [3, 11] to kidney stone formation. On the other hand, ketosis boosts the immune response and has anti-inflammatory and anti-infectious effects [3, 18, 25, 33, 48]. Furthermore, it exhibits a neuroprotective function [3, 18, 25, 33, 48]. The mechanisms underlying neuroprotection are largely unknown. They have been related directly to reduced glucose, elevated fatty acid levels, and β-hydroxybutyrate per se [18]. Further factors might include the modulation of mitochondrial activity, potassium channels, brain-derived neurotrophic factor expression, and neuroinflammation [3, 18, 25, 33, 48]. Ketosis is also associated with a remodeling of the intestinal microbiota. It has been therefore suggested that some benefits of ketosis are mediated by this mechanism [49].

**Table 1:** Direct and indirect effects of ketosis

- Diminished appetite, nausea, vomiting, stomach pain
- Flu-like symptoms (“keto flu”)
- Anti-inflammatory effect
- Anti-infective effect
- Tendency towards metabolic acidosis
- Polyuria (secondary to osmotic diuresis)
- Renal stone formation*
- Phosphate, potassium, and magnesium depletion** (thiamine deficiency)
- Intestinal microbiota remodeling

*Secondary to acidosis, hypercalciuria, and especially hypocitraturia

**Mainly intracellular

In subjects with long-lasting ketosis [50], insulin deficiency (together with increased glucagon secretion) results in depletion of total body phosphate, potassium, and magnesium (regardless of the circulating level), often associated with thiamine deficiency (Table 1).

## Metabolic acidosis in eu- or hypoglycemic ketosis

Acetoacetic acid and β-hydroxybutyric acid dissociate almost completely at the pH of the body fluids. Hence, ketosis causes hypobicarbonatemia and, therefore, acidosis, when acetoacetic and β-hydroxybutyric acids are synthetized faster than they can be excreted and HCO_3_^−^ is metabolized to H_2_CO_3_ and, subsequently, CO_2_ [3, 11, 15, 16]. The anion gap is characteristically increased and the blood lactate concentration normal in ketoacidosis. The anion gap is calculated from the difference between the sum of the major measured cations (Na^+^  + K^+^) and that of the major measured anions (Cl^−^  + HCO_3_^−^). In ketosis, metabolic acidosis (usually defined as pH ≤ 7.35 and HCO_3_^−^  ≤ 20 mmol/L) occurs in the presence of a β-hydroxybutyrate level of about 4.0 mmol/L. However, Kussmaul breathing pattern, fruity acetone odor, relevant hypobicarbonatemia (HCO_3_^−^  ≤ 18 mmol/L), and high anion gap occur with a β-hydroxybutyrate level ≥ 6.0 mmol/L [9, 11, 12].

Acidosis-associated decrease in urinary citrate levels likely underlies the tendency to kidney stone formation [3, 11].

## Diagnostic approach

The presentation of eu- or hypoglycemic ketosis (and ketoacidosis) lacks distinctive features and routine laboratory findings (Table 2). Therefore, its identification is difficult and mandates a high level of suspicion. In contrast to diabetic ketoacidosis [1, 15], patients with eu- or hypoglycemic ketoacidosis are usually lucid and alert despite severe ketoacidosis (except if associated with relevant hypoglycemia). A plausible explanation for this difference [1, 15, 51] is that the neurologic features of diabetic ketoacidosis are largely due to a marked rise in blood tonicity.^2^ This factor is not present in eu- or hypoglycemic ketoacidosis.

**Table 2:** Causes and risk factors for eu- or hypoglycemic ketosis

• Physiological causes
- Stages of life with high rate of glucose utilization (accelerated ketosis): neonatal age, infancy, childhood (≤ 7 years of age), pregnancy, parturition, lactation
- Prolonged exercise
• Further causes
- Binge ethanol ingestion
- Starvation
- Cyclic vomiting syndrome
- Diets low in sugars (or proteins)
- Rare causes: congenital disorder of intermediary metabolism, hyperthyroidism, poisoning (e.g., isopropanol, salicylates), gliflozins

Eu- or hypoglycemic ketosis and ketoacidosis usually occur in association with more than one predisposing factor. For example, (a) infection in a stage of life characterized by accelerated ketosis, (b) preoperative starvation in a diabetic patient treated with a gliflozin, or (c) recent binge ethanol ingestion in a patient with history of undernutrition. Ketosis and ketoacidosis may also occur in subjects with a single predisposing factor such as severe long-lasting starvation. However, severe ketosis more frequently occurs if long-lasting starvation is complicated by an infection. Kussmaul breathing pattern or odor of acetone further support the suspicion.

After a careful history and physical examination, the laboratory work-up includes the urinary nitroprusside-glycine dipstick, the determination of inflammatory markers, blood glucose, and an extended “electrolyte-spectrum” in blood: pH, pCO_2_, HCO_3_^−^, Na^+^, K^+^, Cl^−^, Ca^++^ (either total or ionized), Mg^++^, inorganic phosphate, total protein level (or albumin), uric acid, urea, and creatinine. The measurement of L-lactate, currently integrated in many blood gas analyzers, is helpful to exclude L-lactic acidosis. In this setting, the diagnosis of eu- or hypoglycemic ketosis is confirmed by detecting ketone bodies and that of ketoacidosis by detecting also a metabolic acidosis with an elevated anion gap. The direct determination of circulating β-hydroxybutyrate should facilitate more appropriate clinical decision-making [11, 14, 15].

## Management

### Mainstay

Eu- or hypoglycemic ketosis and ketoacidosis are characterized [3, 11, 15, 16] by (a) poor insulin levels (associated with excess of glucagon) and (b) fluid volume depletion secondary to urinary salt loss with β-hydroxybutyrate (and acetoacetate) to maintain electroneutrality. Poor fluid intake, vomiting, diarrhea, and diaphoresis often play an even more important role than urinary loss in the development of volume depletion.

Hence, oral (cases with ketosis unassociated with relevant acidosis) or parenteral (cases with relevant acidosis) sugar administration increases insulin and reduces glucagon while the fluid administration corrects volume depletion (thereby increasing ketonuria).

In children and adolescents affected by a clinically relevant eu- or hypoglycemic ketoacidosis, a parenteral hydration with isotonic saline supplemented with 5% dextrose (it contains approximately dextrose 50 g/L, sodium 154 mmol/L, and chloride 154 mmol/L) or lactated Ringer and 5% dextrose (it contains sodium 130 mmol/L, potassium 4 mmol/L, calcium 1.4 mmol/L, chloride 109 mmol/L, lactate 28 mmol/L, and dextrose 50 g/L) is advised [52, 53]. However, the rates of glucose and saline infusion must be adjusted based on clinical and laboratory findings [52, 53]. Furthermore, since alcoholic ketoacidosis occasionally presents with overt hyperglycemia, insulin administration rather than glucose may sometimes be required. Finally, parenteral glucose should be initially avoided in the presence of severe hypokalemia since it induces insulin secretion, which drives potassium into the cells thereby worsening hypokalemia [52].

Ondansetron, an antagonist of the type 3 serotonin receptor, is also advised in children with cyclic vomiting syndrome [54]. Like for diabetic ketoacidosis, there is no role for HCO_3_^−^ as a therapy for eu- or hypoglycemic ketosis and ketoacidosis [52].

### Refeeding syndrome and thiamine deficiency

In subjects with long-lasting ketosis, insulin deficiency results in a total body depletion of phosphate, potassium, and magnesium (regardless of the circulating level). The sugar delivery as part of the treatment strategy is followed by an increased circulating insulin level that induces a rapid uptake of phosphate, potassium, and magnesium into cells with subsequent fall of the blood concentration of these ions [50]. In addition, the body begins to retain fluids, and the extracellular space expands. The most effective way to treat these electrolyte and fluid abnormalities, which have been called “refeeding syndrome,” is to be aware of them. One should start feeds slowly and aggressively supplement and monitor phosphate, potassium, and magnesium for approximately 4 days after feeding is started [50]. Because alcohol dependence is often associated with thiamine deficiency, it is also advised to parenterally administer 100 mg of thiamine in ketoacidosis after binge ethanol ingestion [15, 42].

## Conclusions

Although the existence of ketone bodies has been known since the nineteenth century, the role of these metabolites has often been overlooked outside of diabetes mellitus. However, over recent years it has become evident that ketone bodies are important in various clinical conditions. In the context of acid–base balance evaluation, it is advisable to routinely integrate, in addition to pH, pCO_2_, HCO_3_^−^, Na^+^, K^+^, and Cl^−^, the assessment of lactic acid and, if possible, β-hydroxybutyrate.

## Data Availability

Not applicable.

## Footnotes

1 At increasing concentration of ketone bodies, the ratio of hydroxybutyrate to acetoacetate ratio, which is normally 1:1, increases to 5:1 in diabetic ketoacidosis or even to 10:1 in acidosis after binge ethanol ingestion.

2 The blood tonicity, also known as effective blood osmolality, denotes the concentration of sodium, glucose (and mannitol), and solutes that are impermeable to cell membranes and are therefore restricted to the extracellular compartment.
